# Amplification and overexpression of the *ID4 *gene at 6p22.3 in bladder cancer

**DOI:** 10.1186/1476-4598-4-16

**Published:** 2005-05-05

**Authors:** Qiong Wu, Michèle J Hoffmann, Florian H Hartmann, Wolfgang A Schulz

**Affiliations:** 1 Dept. of Urology, Heinrich Heine University, Düsseldorf, Germany

## Abstract

**Background:**

Amplifications at 6p22.3 are prevalent in advanced stage bladder cancer (TCC). Previous studies have identified *SOX4*, *CDKAL*, and *E2F3 *as targets of this amplification and therefore potential oncogenes, but the more telomeric *DEK *gene too has been reported as overexpressed and amplified. We have therefore investigated whether the intermediate region harboring the oncogene candidate *ID4 *is also part of the amplicon.

**Results:**

Expression of *E2F3*, *DEK*, and *ID4 *was investigated by real-time RT-PCR in 28 TCC compared to 6 normal bladder tissues and in 15 TCC cell lines compared to cultured normal urothelial cells. Expression of *E2F3 *as well as *DEK *increased on average in tumor vs. normal tissues (3-fold and 2.5-fold, resp.), but only the increase for *E2F3 *was statistically significant (p = 0.039). ID4 overexpression was observed in selected specimens. Each of the three genes was overexpressed in several cell lines, up to 150-fold (*ID4*), 30-fold (*E2F3*), and 9-fold (*DEK*), but these increases were not correlated to each other. Instead, moderate (*DEK*) to excellent (*ID4*) correlations were observed with copy number increases of microsatellites near each gene. Microsatellite copy number increases were highly heterogeneous across the investigated several Mb region revealing at least three subregions of amplification.

**Conclusion:**

Extending previous reports, our data indicate that the 6p22.3 amplicon in TCC is highly heterogeneous and targets several genes in a variable fashion. Among these, expression of *E2F3 *and *DEK *appear to be generally increased in TCC, with additional increases caused by amplifications. In contrast, over-expression of *ID4*, which is normally predominantly expressed in testes and brain, appears to depend more strictly on gene amplification. Accordingly, the effect of amplifications at 6p22.3 in bladder cancer is expected to be non-uniform, thereby contributing to the highly variable biological and clinical behavior of advanced stage tumors. *ID4 *is a potential oncogene in a small subset of bladder cancers.

## Background

Urothelial carcinoma, which is commonly called bladder cancer, occurs in two forms, a more prevalent papillary subtype and a rarer, but much more invasive subtype [1,2]. Invasive bladder cancers usually develop from highly dysplastic carcinoma in situ, but some papillary tumors also progress to an invasive form. While papillary cancers often contain a limited number of chromosomal alterations, invasive cancers are characterized by a high degree of chromosomal instability [3,4]. Even T1 stage cancers, which have only invaded the lamina propria underlying the urothelium, often exhibit multiple chromosomal changes. Cancers at more advanced stages accumulate further chromosomal alterations. In particular, they harbor amplifications, e.g. of regions from chromosomes 5p, 6p, 8q, 11q, and 20q [4-7]. It is generally assumed that chromosomal segments consistently amplified in a cancer contain oncogenes [8]. Accordingly, genes amplified in advanced bladder cancers would be expected to contribute to the progression of this cancer.

**Figure 1:**
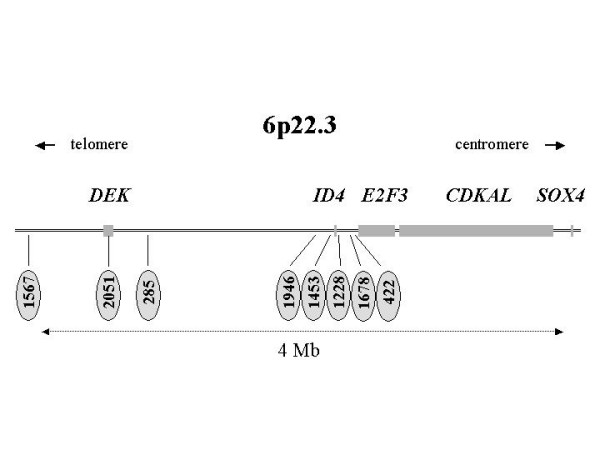
**The chromosome 6p22.3 region**. Verified genes are drawn to size as grey boxes and the location of microsatellites used (the prefix D6S is omitted) is indicated.

One of the most consistently amplified region in advanced bladder cancers is located at 6p22.3 [5-7,9-12]. This amplification is detected in up to 25% of advanced stage bladder cancers and is present in many bladder cancer cell lines. The cell lines harboring this amplification provide a convenient experimental access to map the amplified region precisely and identify potential urothelial carcinoma oncogenes. Mapping of the 6p22.3 amplicon has been performed by several groups who have identified different genes as potential targets of the amplification (Figure 1). In a first study [5], *SOX4 *was identified as a frequent, but not entirely consistent amplification target. Further studies revealed that many amplifications also included *E2F3 *and the encoded protein was over-expressed, particularly in high stage and high grade urothelial cancers [6,11,12]. A high resolution analysis by microarray-based comparative genomic hybridization identified *CDKAL1 *located between *SOX4 *and *E2F3 *as the most commonly amplified gene [7]. Another study indicated that *DEK *located further telomerically (Figure 1) may be amplified in a substantial proportion of bladder cancer tissues [10]. *DEK *was also found to be over-expressed in a cDNA microarray study, albeit predominantly in early stage tumors [12].

This somewhat confusing state may owe partly to the fact that many studies were performed prior to the publication of the finished sequence of chromosome 6 in October 2003 [14] and partly to the use of different techniques. Alternatively, the differences between the studies could also mean that the region of amplification is not uniform and that multiple genes might be targets.

Most previous studies have focussed on a more centromeric region within 6p22.3 containing *SOX4*, *CDKAL1*, and *E2F3 *(Figure 1). *DEK *is located about 2 Mb more telomeric of these genes. The interval framed by *E2F3 *and *DEK *contains another plausible oncogene candidate, i.e. *ID4*. The ID proteins ('inhibitor of differentiation') are named for their ability to bind and inhibit cell-type specific helix-loop-helix transcriptional activators inducing cell differentiation. Accordingly, they tend to stimulate cell proliferation, and have been implicated in various cancers [15-18]. Compared to ID1 and ID2, ID4 is a less well characterized member of the family. It is expressed in a tissue-specific manner, with the highest levels in testes and brain [19]. In the present study, we have therefore investigated to which extent *ID4 *gene copy numbers and expression are affected by 6p22 amplifications in bladder cancer, in comparison to *E2F3 *and *DEK*.

## Results

### Expression of 6p22 genes in bladder cancer cell lines

**Figure 2:**
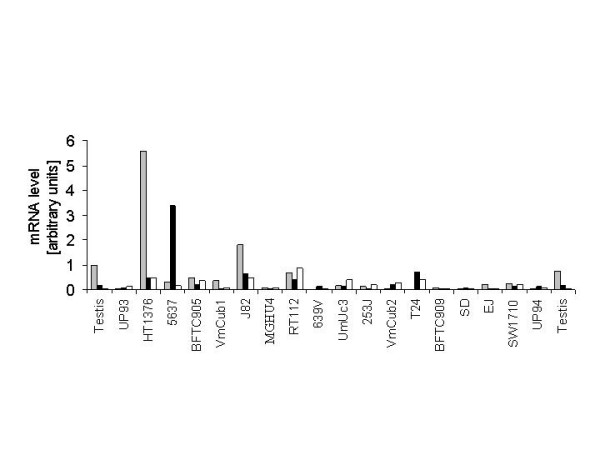
**Expression of 6p22 genes in bladder cancer cell lines**. Levels of mRNAs for ID4 (grey bars), E2F3 (black bars), and DEK (white bars) were determined by real-time RT-PCR relative to β-actin mRNA. As controls, shown to the left and right of the cancer cell lines, two independent primary cultures of normal urothelial cells (UP93 and UP94) and two different normal testicular tissues were used. All values were determined by at least triplicate measurements, with further repeats, if deviations exceeded 20% of the mean. Arbitrary units are given; values for E2F3 and DEK were divided by 10.

First, expression of ID4 mRNA in comparison to E2F3 and DEK mRNAs was investigated by real-time PCR in 16 TCC cell lines (Figure 2). Normal urothelial cells (UP) proliferating in culture and testicular tissue samples served as controls. Expression at least twice as strong as in normal urothelial cells was considered as over-expression. According to this criterion, twelve cell lines over-expressed *ID4*, with a maximum >150fold increase in HT1376. In 6 cell lines, *E2F3 *was over-expressed. The *E2F3 *over-expressing cell lines included HT1376 and 5637 in line with previous reports [7,11]. 5637 displayed a >30fold increased level of E2F3 mRNA. Six cell lines over-expressed *DEK*, although the relative increases were in general more moderate, with a maximum 9fold increase in RT112. Inspecting Figure 2 may suggest that cell lines over-expressing one gene also tended to over-express one or both others. However, this tendency was not reflected in a statistically significant correlation. Specifically, expression did not significantly correlate for any pair of genes, the best correlation coefficient reaching 0.39 between *ID4 *and *DEK*. The divergence is strikingly illustrated by the cell lines HT1376 and 5637, which presented increased expression levels for all three genes, but with either *ID4 *or *E2F3 *displaying particularly pronounced increases (Figure 2).

### Gene amplification analysis

**Figure 3:**
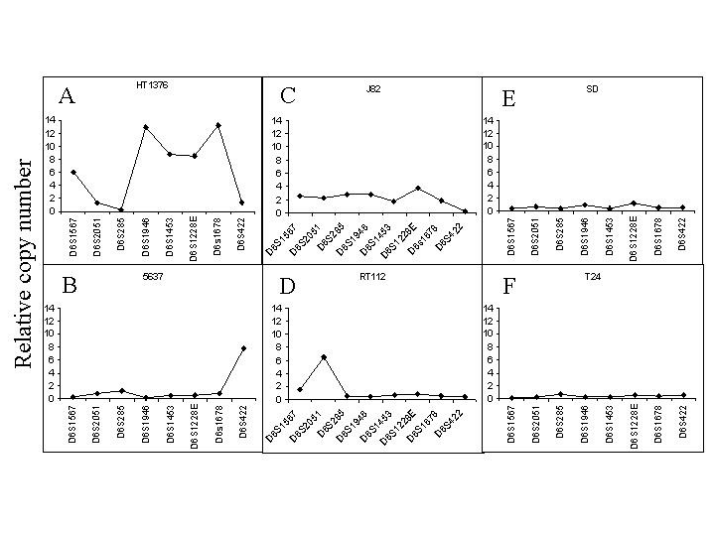
**Copy numbers of 6p22 microsatellites in bladder cancer cell lines**. Microsatellite copy numbers (see Fig. 1 for their location) were determined as described in the methods section in the cell lines (A-F) HT1376, 5637, J82, RT112, SD, and T24. Normal genome equivalents determined from leukocyte DNA were set as 1.

To determine whether the increases in mRNA expression were due to gene amplification, copy numbers were investigated for eight microsatellite loci located in 6p22.3. Five were located around *ID4*, from D6S422 close to *E2F3 *to DS1946, and three were located around *DEK*, including the intragenic marker D6S2051 (Figure 1). The results (Figure 3) demonstrate a considerable variation in the microsatellite copy numbers across the region, even within the same cell line. For instance, in HT1376 the copy numbers of the eight microsatellites ranged from approximately 0.5 to 13 normal genome equivalents, which would correspond to 1 – 26 copies in a diploid cell. As all bladder cancer cell lines are aneuploid, typically hypo- or hypertriploid with a modal distribution, between 2 – 40 copies would be present in a single cell. In HT1376 specifically, two different segments of amplification are discernible, one telomeric to *DEK *and one around *ID4*. In fact, amplification of the region including *E2F3 *and of *SOX4 *has been shown previously in this line [7,9,11]. Thus, there are at least three distinct regions of amplification in this cell line. In contrast, no amplification of microsatellites telomeric of D6S422 was evident in 5637, which contains increased copy numbers of *E2F3 *and *SOX4 *[7,9,11]. A more homogeneous increase of all more telomeric markers including those close to *ID4 *and *DEK *was seen in J82. RT112 contained a selective amplification of the D6S2051 marker located in the *DEK *gene in accord with the maximum expression of this gene in this cell line. None of the microsatellites showed increased copy numbers in T24 or SD, although *E2F3 *as well as *DEK*, but not *ID4 *were overexpressed in T24.

**Figure 4:**
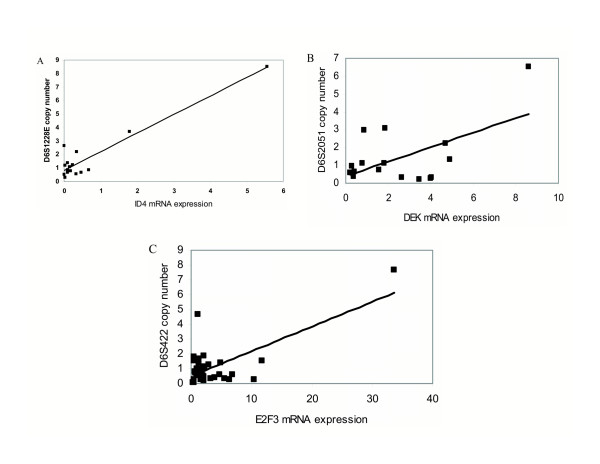
**Relationship of 6p22 gene expression changes to copy number changes of adjacent microsatellites in bladder cancer cell lines**. A: ID4 mRNA vs. D6S1128E (r = 0.95), B: DEK mRNA vs. D6S2051 (r = 0.57), C: E2F3 mRNA vs. D6S422 (r = 0.79),

Expression of *ID4 *correlated excellently with the copy numbers of each of the microsatellites around the gene, yielding coefficients between 0.89 and 0.95 (Figure 4A). A closer inspection shows however that this strong correlation is primarily caused by the cell lines with clear-cut amplifications of the region, i.e. J82 and HT1376 while at lower copy numbers the relationship is essentially random. *DEK *expression correlated moderately well with the copy number of the intragenic D6S2051 marker (Figure 4B). *E2F3 *expression showed the best correlation with the microsatellite marker D6S422 located most closely to the gene (Figure 4C). However, this apparent correlation was mostly due to the strongly increased copy number of D6S422 in the 5637 cell line; if this data point is removed, the relationship is essentially random.

### 6p22 gene expression in bladder cancer tissues

**Figure 5:**
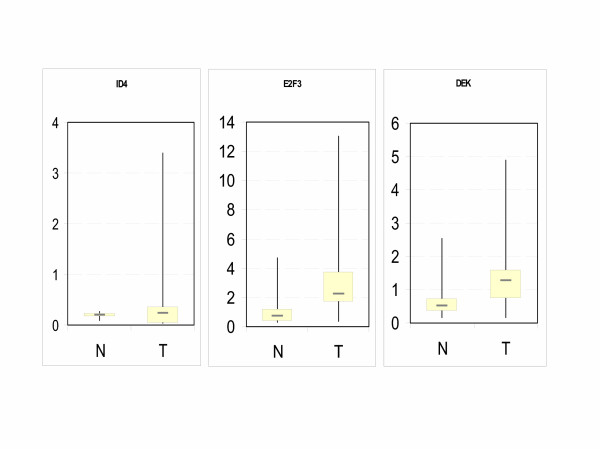
**Expression of genes at 6p22.3 in bladder cancer tissues**. Box plot representation of ID4 (left), E2F3 (center), and DEK (right) mRNA expression relative to β-actin mRNA as determined by real-time RT-PCR in 28 bladder cancer tissues (see Table 1) and 6 morphologically normal bladder tissues. Note the different scales in the three figure parts.

To determine whether the results from the cell lines can be extended to bladder cancer tissues, the expression of the three genes was determined in 28 tumor tissue samples and 6 morphological normal samples from cystectomy specimens by real-time RT-PCR (Figure 5, Table 1). In accord with previous reports, expression of E2F3 mRNA and DEK mRNA were often increased in tumor compared to normal tissues. Median E2F3 mRNA expression was 2.24 arbitrary units in cancers compared to 0.72 in normal tissues, i.e. about threefold higher. This difference was statistically significant (p = 0.039). Median DEK mRNA expression was 1.26 compared to 0.52, i.e. 2.5fold higher, but the difference did not reach statistical significance. In contrast, ID4 expression essentially did not differ between normal and cancer tissues. Instead, individual cancer specimens showed strongly increased expression beyond the range of normal tissues.

**Table 1:** Tumor tissues investigated

	**Patient**		**Tumor**				**mRNA expression (AU)**		
**No**	**Sex**	**Age**	**Stage**	**Lymph node status**	**Metastasis status**	**Grade**	**E2F3**	**ID4**	**DEK**

3	m	75	pT3b	N0	M0	G2	5.59	0.48	4.88
6	m	76	pT3b	pN2	M0	G3	10.37	0.36	1.37
12	m	70	pT3a	N0	M0	G3	1.73	0.04	1.99
28	m	72	pT3b	N0	M0	G3	3.73	0.24	2.27
41	f	54	pTa	Nx	Mx	G2	11.69	0.49	3.29
47	m	76	pT3b	N0	M0	G3	3.75	0.19	1.84
52	f	87	pT3b	N0	M0	G3	2.86	0.25	1.43
55	m	83	pT4a	N1	M0	G3	1.25	0.18	0.52
61	m	75	pT3b	N0	M0	G3	1.98	0.12	1.48
62	f	78	pT3b	N0	M0	G2	1.60	0.54	0.72
64	m	74	pT3b	N2	M0	G3	3.34	0.11	1.26
67	f	94	>pT2	Nx	Mx	G3	4.38	0.33	1.50
69	m	77	pT3b	N0	M0	G3	2.17	0.14	1.19
104	m	61	pT1	N0	M0	G2	1.49	0.24	0.72
105	m	83	pTa	Nx	M0	G2	3.80	0.27	1.88
109	m	68	pT4a	N0	M0	G3	2.14	0.23	0.30
111	m	81	pT3b	N0	M0	G2-3	2.24	0.52	0.61
115	m	74	pT3a	N2	M0	G3	1.74	0.12	0.84
120	m	66	pT2	N0	Mx	G2	3.12	0.15	0.66
150	f	78	pT3a	N2	M0	G3	0.54	0.20	0.86
168	m	64	pT3a	N2	M0	G3	1.68	0.12	1.42
170	f	63	pT2	N0	M0	G2	0.58	0.08	0.27
172	m	72	pT3a	N0	M0	G3	1.73	0.13	0.85
205	f	65	pT2	N0	M0	G2	0.36	0.10	0.17
212	m	73	pT2a	N1	M0	G2	0.62	0.16	0.79
224	m	65	pT2a	N0	M0	G2	4.29	3.39	1.87
231	m	95	>pT2	Nx	M0	G2	13.04	1.11	1.59
246	m	67	pT4a	N2	M0	G3	3.07	0.27	1.29

## Discussion

Taken together with previous analyses of 6p22.3 amplifications in bladder cancer, the present study has implications concerning the structure of 6p22.3 amplicons, the effect of the amplification on gene expression, and more generally, concepts of the significance of gene amplification in human cancers. Specifically, the findings raise interesting aspects with regard to *ID4*.

Our findings indicate that 6p22.3 amplifications in bladder cancer are even more heterogeneous than hitherto assumed. Previous studies have identified the centromeric region around *CDKAL1 *as part of an amplicon that often, but not consistently included *SOX4 *and *E2F3 *[7,9,11,12]. The telomeric region around *DEK *had not been investigated as well yet [10]. Our study confirms that this region is also subject to copy number gains and amplifications. Specifically, our findings are in good accord with ref. [10] describing frequent copy numbers gains of microsatellites around *DEK*, but at highly variable frequencies. The three markers investigated in that study were located within 0.5 Mb and were amplified contiguously in 24% of the specimens, whereas each of these three markers individually was amplified in 45%, 56%, and 64% of the cases. Thus, upon closer analysis, this region of amplification appears also heterogeneous in itself. The intermediate region harboring *ID4 *had been more or less disregarded in previous studies, but our data indicate that it is clearly gained or even amplified in a certain number of cases, sometimes concomitantly with, and sometimes independent of the other two regions. In summary, therefore, one might discern three main segments of amplification, which split up into further subregions in individual cancers. The 6p22.3 amplicon therefore seems to belong to a class which is characterized by pronounced heterogeneity and great structural complexity (see below).

Previous studies have variously identified *SOX4*, *E2F3*, *CDKAL1*, and *DEK *as potential targets of the 6p22.3 amplification; the present study adds *ID4 *to this list. In this regard, it is interesting to compare the cell line data, where *ID4 *emerged as the most frequently over-expressed gene with the tissue data which showed generalized increases in expression of *E2F3 *and of *DEK *(Figure 1 vs. Figure 5). This apparent discrepancy can be relatively simply resolved by two plausible assumptions. Normal bladder tissue is largely quiescent, albeit proliferation increases strongly in response to tissue damage [20]. Thus, urothelial cancers are distinguished from normal tissue not only by expression of cancer-specific genes, but also by generalized over-expression of genes associated with cell proliferation. The generally increased expression of *E2F3 *and *DEK *in the cancer tissues may reflect the latter effect, with further increases in individual cases due to deregulation and copy number gains of these genes. In contrast, early passage cultured urothelial cells proliferate as rapidly as cancer cell lines [21-23]. Therefore, the increases in *E2F3 *and *DEK *in cultured cancer vs. normal cells may turn out as comparatively moderate. In contrast, *ID4 *expression has probably to be considered as ectopic in bladder cancer, since it is normally restricted to other tissues including testes and brain [19]. Thus, overexpression may more strictly depend on amplification of the gene, particularly in tumor tissues.

It is commonly assumed that regions in the genome that are amplified in cancers harbor proto-oncogenes that are activated by overexpression as a consequence of increased gene dosage. Indeed, several bona fide oncogenes have been found in amplified regions and some have even been identified by cloning from amplicons. In such cases, amplicons consistently contain one particular gene, alone or together with a limited number of others, e.g. *ERBB2 *only or together with *TOP2A*. Interestingly, the structure of such amplicons can be quite simple [24-26]. The mechanism underlying such amplifications is not understood in detail, but appears to involve the re-replication of a single chromosome fragment, most likely via a circular double-minute intermediate [26].

Clearly, the 6p22 amplicon in bladder cancer belongs to a different class of amplicons which are characterized by great heterogeneity and instability. Such amplicons often contain different segments and accordingly different genes from one region and even sequences from different chromosomes [27-30]. The mechanism causing these amplifications is considered to be most likely breakage-fusion-bridge cycles initiated e.g. by hypoxia [31] or breakage at fragile sites [32].

Considering this background, the question which is the oncogene targeted by 6p22 amplifications in bladder cancer and the specific issue of the role of *ID4 *have to be approached with due caution, since in each individual case the amplicon may be influenced by random factors such as the location of an initiating double-strand break and structural factors such as preferred sites of breakage of dicentric chromosomes arising during breakage-fusion-bridge cycles. Nevertheless, the relatively high prevalence of 6p22 amplifications in bladder cancer and the relative specificity of this amplification for this cancer type argue for a functional selection as well.

For several 6p22 genes subject to amplification, it is plausible to assume that their overexpression may confer a more aggressive phenotype to bladder cancer cells. SOX factors determine cell fate and cell differentiation [33], so *SOX4 *overexpression might lead to further dedifferentiation. E2F transcription factors activate the transcription of genes required for DNA synthesis and E2F3 appears to repress some promoters that are activated by E2F1, including the ARF promoter in *CDKN2A *[34]. DEK is part of a oncogenic fusion protein resulting from t(6;9)(p23;q34) translocations in acute myeloid leukemia [35] and is implicated in regulation of chromatin structure, which is evidently aberrant in invasive bladder cancers. However, specific functional studies on the role of these proteins in urothelial cells are lacking. The function of CDKAL1 is entirely unknown.

ID4 belongs to a protein family whose members have been shown to interfere with cell differentiation by blocking the effects of HLH transcription factors and pocket proteins, including RB1. Several members have been reported to be over-expressed in human cancers [15-18]. *ID4 *in particular has been shown to be overexpressed in rat mammary carcinomas. Accordingly, overexpression of ID4 blocked the differentiation of HC11 mammary epithelial cells and stimulated their proliferation [36]. It is also the target of a specific chromosomal translocation in some cases of B-cell acute lymphoblastic leukemia [37]. Conversely, ID4 expression has been reported to be down-regulated by promoter hypermethylation in colon carcinomas [38]. Evidently, as for the other oncogene candidates on 6p22, more detailed studies are required on the biochemical and functional properties of ID4 in normal and cancerous urothelial tissue.

## Conclusion

In conclusion, our study indicates that the 6p22.3 amplification prevalent in advanced bladder cancers is highly heterogeneous and contributes to the altered expression of several genes, including *ID4*, in a highly variable manner. Thus, this genetic change may contribute to the highly variable biological and clinical behaviour of invasive bladder cancers.

## Methods

### Tissues

Cancerous and normal bladder tissues were used from a previous study [39]. Normal tissues were identified by gross morphology, with microscopic verification in case of extended tumors. Important clinical parameters of the cancer tissues are summarized in Table 1. RNA from testicular normal and cancer tissues used as a control for ID4 expression was also prepared in the course of a former study [40].

### Cell lines and primary cultures

The bladder cancer cell lines 253J, 639v, 5637, BFTC905, BFTC909, EJ, HT1376, J82, MGHU4, RT112, SD, SW1710, T24, UMUC3, VMCub1, and VMCub2 and primary urothelial cells were cultured as described previously [23].

### DNA and RNA extraction

High-quality DNA and RNA were extracted by standard methods using commercial kits from Qiagen (Hilden, Germany) and Peqlab (Erlangen, Germany).

### RT-PCR

Following photometric quantification, 2 μg mRNA were transcribed into first strand cDNA using SuperscriptII (Invitrogen, Karlsruhe, Germany) according to the manufacturer's protocol with oligo-dT primers. Real-time RT-PCR was carried out using a LightCycler instrument (Roche Diagnostics, Mannheim, Germany) and the primers indicated in Table 2. The amplification mixture consisted of 1x reaction mix (LightCycler-FastStart DNA Master PLUS SYBR Green I; Roche Diagnostics), 10 pmoles (*DEK*, *E2F3*, *ID4*) or 5 pmoles (β-actin) of each primer and 20 ng cDNA in a final volume of 10 μl. The reaction was monitored between the annealing and elongation steps at 640 nm. After the final cycle, melting-point analysis of the samples was performed over the range of 69°C – 99°C. Turning-point values for the specific genes were related to those for β-actin.

**Table 2:** RT-PCR primers and conditions

Designation	Sequence	T_m_(°C)	Product size (bp)
GAPDH 350s	TCCCATCACCATCTTCCA	62.3	379
GAPDH 350as	CATCACGCCACAGTTTCC	61.9	
Aktin661S	TGACGGGGTCAC	72.2	661
Aktin661AS	CTAGAAGCATTT	70.9	
ID4 fw	CCGCCCAACAAGAAAGTCAG	59.4	188
ID4 rv	GGTGTTGAGCGCAGTGAG	58.2	
E2F3 fw	ACGTCTCTTGGTCTGCTCAC	59.4	155
E2F3 rv	TCTTAATGAGGTGGATGCCT	55.3	
DEK fw	GTGGGTCAGTTCAGTGGC	58.2	291
DEK rv	AGGACATTTGGTTCGCTTAG	55.3	

### Microsatellite analysis

Microsatellites located at 6p22 (see Figure 1) were amplified using published primer sets (see the ensembl database for sequences and T_m_s) in duplex reactions with one microsatellite from chromosome 12 (D12S1650) or chromosome 15 (D15S127) as control. These chromosome are rarely affected by allelic imbalances in bladder cancer [3]. One primer from each pair was labeled with IRD-800 fluorescence and the products were resolved and detected on a LiCOR 4200S automated sequencer. Reactions were carried out in the linear phase of PCR with 25–30 cycles, the precise number being determined for each primer pair. Band intensities were quantitatively determined by ONE-D-SCAN (MWG-Biotech, Ebersberg, Germany). Leukocyte DNA standards were included in each set of reaction. The ratio of intensities of chromosome 6 and chromosome 15 microsatellites in these was set as 1 for each pair.

## Authors' contributions

QW performed most experiments and most of the data evaluation, aided and supported by MJH; FHH contributed and evaluated the clinical data; WAS conceived and supervised the study and drafted the manuscript.
